# Transcriptome analysis of cynomolgus macaques throughout their lifespan reveals age-related immune patterns

**DOI:** 10.1038/s41514-024-00158-0

**Published:** 2024-06-20

**Authors:** Hyeon-Mu Cho, Se-Hee Choe, Ja-Rang Lee, Hye-Ri Park, Min-Gyeong Ko, Yun-Jung Lee, Hwal-Yong Lee, Sung Hyun Park, Sang-Je Park, Young-Hyun Kim, Jae-Won Huh

**Affiliations:** 1 National Primate Research Center, Korea Research Institute of Bioscience and Biotechnology (KRIBB), Cheongju, 28116 Republic of Korea; 2 Department of Functional Genomics, KRIBB School of Bioscience, University of Science & Technology (UST), Cheongju, 28116 Republic of Korea; 3 Primate Resources Center, Korea Research Institute of Bioscience and Biotechnology (KRIBB), Jeongeup, 56216 Republic of Korea

**Keywords:** Genome, Transcription

## Abstract

Despite the different perspectives by diverse research sectors spanning several decades, aging research remains uncharted territory for human beings. Therefore, we investigated the transcriptomic characteristics of eight male healthy cynomolgus macaques, and the annual sampling was designed with two individuals in four age groups. As a laboratory animal, the macaques were meticulously shielded from all environmental factors except aging. The results showed recent findings of certain immune response and the age-associated network of primate immunity. Three important aging patterns were identified and each gene clusters represented a different immune response. The increased expression pattern was predominantly associated with innate immune cells, such as Neutrophils and NK cells, causing chronic inflammation with aging whereas the other two decreased patterns were associated with adaptive immunity, especially “B cell activation” affecting antibody diversity of aging. Furthermore, the hub gene network of the patterns reflected transcriptomic age and correlated with human illness status, aiding in future human disease prediction. Our macaque transcriptome profiling results offer systematic insights into the age-related immunological features of primates.

## Introduction

Aging, commonly considered a chronological time passage, a risk factor for increasing morbidity and mortality rates^1,2^. Recent technological advances in disease management have amplified interest in improving the quality of life, leading to an increase in life expectancy. Biological age, as opposed to chronological age, is measured using biometrics or biomarkers, and reflects the real physiological state of aging, enabling health span calculation^3^. Recent studies from various fields, including genomics, transcriptomics, epigenetics, and proteomics, have identified several aging-related biomarkers, biological mechanisms of aging, and biological age predictors based either on transcriptomics, epigenetics, or both^4–6^. Epigenetic signatures, which are widely studied biomarkers of aging, are directly affected by environmental factors and regulate gene transcript expression via chemical modification of certain DNA positions^7,8^. Therefore, both transcriptome and the epigenome, broadly utilized in recent biological studies, are vulnerable to environmental exposure, which contributes to disease progression^9^. In most human aging research, cross-sectional data from different age groups are analyzed together, despite the cumulative effects of different environments over the aging process. Controversial results may arise from the distorted omics data due to poor sampling. However, animal models such as yeast, worms, flies, fish and rodents, offer more control over environmental factors, allowing researchers to discover various factors influencing both acceleration and intervention of aging^1,10^. However, in human biomedical research, more complex models that closely resemble humans, encompassing physical, genetic, and social aspects, are necessary. Non-human primates (NHPs) are promising aging models for overcoming the difficulties mentioned^10,11^.

Alterations in the immune system are currently the subject of lively debate in aging research. The chronic low-grade inflammation caused by activation of innate immunity is a crucial phenomenon that occurs with aging and is globally known as “inflammaging”^12,13^. Additionally, impaired function of immunity changes in older individuals, known as “immunosenescence”, prompt susceptibility to infectious or age-related diseases, damaging the overall biological system of the body and accelerating their biological age^14–16^. Epigenetic factors have recently been regarded as mediators between aging and immune response. Shchukina et al. focused on multi-omics data of immune cells and found age-dependent hypomethylation of DNA, which is associated with upregulated genes during aging. Urban et al. suggested that aging-induced hypomethylated DNA triggers pattern recognition receptor (PRR) signaling, deteriorating inflammatory dysfunction^17,18^. These epigenetic changes in DNA sequences indicate transcriptomic modifications via differential gene expression or alternative splicing^19^.

We investigated the transcriptomic features of healthy and specific pathogen-free cynomolgus macaques (*Macaca fascicularis*). To explore whole lifespan, eight male macaques were divided into four age group each containing two individuals. As a laboratory animal, the macaques were protected from all environmental factors other than aging. Three years of this study revealed immune-related gene expression patterns and three primary patterns were identified. The increased pattern was mostly associated with innate immune cells prompting inflammaging, and the other two decreased patterns were associated with the down-regulation of adaptive immune cells, accelerating immunosenescence. We also found that the group of genes characterized by these patterns was compatible with the gene expression patterns in human blood. The identified characteristics of the whole transcriptome analysis will facilitate further discovery of aging candidate genes and potential network pathways of aging.

## Results

### Considerable DEGs during puberty and specific isoform changes during old age

**Fig. 1:**
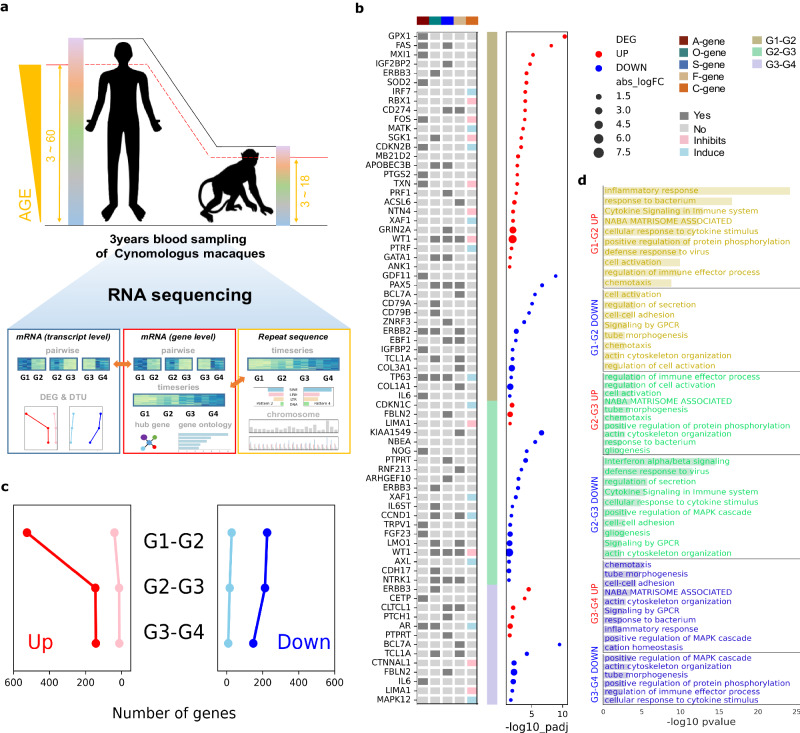
Pairwise analysis of RNA sequencing data. **a** Three years of annual whole blood sampling of *Macaca fascicularis*. Age group 1 to 4 of 8 individuals with age ranging from 3 to 18 which is equivalent to approximately human age 3 ~ 60. RNA sequencing and small RNA sequencing with various downstream analysis including adjacent age group pairwise and time-series analysis coupled with interspecies data comparison with human. mRNA transcript, transposable elements transcript and miRNA transcript were analyzed. In the case of mRNA, gene level and transcript level count were separately examined. 3’UTR length of the gene was also analyzed in relation to the interaction with small RNA. **b** Annotated information of DEGs from each age stage. A-gene: aging genes, O-gene: oncogene, S-gene: tumor suppressor gene, F-gene: fusion gene, C-gene: cell-age gene. A- ang C-gene information were from GenAge database and O-, S- and F-gene are from Cancer Census database. Inverse log p value of adjoining pairwise DEG analysis are visualized with up DEG colored red and down DEG colored blue. **c** The count number of differentially expressed genes (DEGs) from adjoining group pairwise analysis and differential transcript usages (DTUs) from isoform switch analysis following pseudoalignment by Kallisto on each age stage. Red, pink, blue and skyblue indicate up DEGs, up DTUs, down DEGs and down DTUs respectively. DEG count (G1-G2 up,down: 525,225; G2-G3 up,down: 146,213; G3-G4 dp,down: 143,148) DTUcount (G1-G2 up,down: 46,37; G2-G3 up,down: 18,21; G3-G4 dp,down: 17,13). **d** Identified GO terms of up and down DEGs from 3 age stages by Metascape. Source data are provided as a Source Data Fig. 1 file.

To explore the wide range of lifespans, we divided cynomolgus macaques into four age groups (G1, G2, G3, and G4) with two individuals in each group (Supplementary Table 1). For stringent analysis, laboratory macaques have been housed under identical conditions, affected by the same environmental factors, including diet, temperature, or background stresses throughout their lifetime^12^. Three years of annual peripheral blood sampling provided 24 samples ranging from 3 to 18 years of age, equivalent to a human age of 3-60 years (Fig. 1a, and Supplementary Fig. 1a). RNA sequencing and subsequent batch-effect correction were performed to obtain more reliable results (Supplementary Fig. 1b). First, a pairwise comparison analysis was performed to identify differentially expressed genes (DEGs) between the two nearby groups. The number of upregulated and downregulated DEGs between G1 and G2 was 525, 225 respectively, which was more than twice the number of the other two pairwise comparisons (Fig. 1c, Supplementary Fig. 1e). Given that the sexually mature age of macaques is approximately 4-6 years old^20^, the noteworthy differentiation between G1 and G2 is reproductive capacity, representing puberty. To identify the features of the genes, we cross-checked them with both gene lists from cancer census genes (CGC) from the COSMIC^21^ and GenAge databases^22^ (Fig. 1b). *ERBB3*, a oncogene in CGC, was detected as one of the 21 genes that belonged to all three pairwise comparisons (Fig. 1b, Supplementary Fig. 1c, d). This gene is essential for cell growth, activating the PI3K/AKT/mTOR pathway^23–25^. As this feature plays a critical role in body development, increasing the expression pattern of the gene in stage G1-G2 is a prerequisite for puberty. However, another increasing pattern of the gene in stage G3-G4 may have a detrimental effect on aged individuals, as its overexpression activates malignant tumor growth^26^ (Supplementary Fig. 1f). Gene ontology (GO) analysis was performed to characterize DEGs using Metascape. Upregulated and downregulated DEGs at each stage were analyzed and the immune-related terms comprised the majority of the GO results (Fig. 1d). The most significant changes across all stages were observed in the upregulated DEGs of G1-G2. These genes were predominantly enriched in GO terms such as “inflammatory response,” “response to bacterium,” “Cytokine Signaling in Immune System,” and “defense response to virus.” Additionally, the GO term “response to bacterium” was associated with upregulated DEGs in both G2-G3 and G3-G4, whereas the terms “Interferon alpha/beta signaling,” “defense response to virus,” and “Cytokine Signaling in Immune System” were related to downregulated DEGs in G2-G3. These GO results were in line with previous studies showing that the immune system at an early stage of life strongly respond to novel pathogen infection and subsequently declines with aging, especially in viral infections^27–30^.

In addition, transcript-level pairwise comparisons of the age groups were performed to better understand aging. A different sequencing data analysis pipeline was used to detect transcript-level read counts, in contrast to the gene-level DEG analysis mentioned earlier. Differential transcript usage (DTU) was calculated to determine the changes in different transcript expression ratios within the same gene. The number of upregulated and downregulated DTUs between G1 and G2, representing puberty, was 46 and 37, respectively, which was higher than that of the other two stages (Fig. 1c, Supplementary Fig. 2a). We identified 16 DTUs that covered more than two stages of transcript alteration, and cross-checked all DTUs with the detected DEGs (Supplementary Fig. 2b, Supplementary Fig. 2d). The expression of *CTSW*, which encodes Cathepsin W, significantly fluctuated at both the transcript and gene levels (Supplementary Fig. 2c). The gene was downregulated and upregulated at the G1-G2 and G2-G3 stages, respectively. At transcript level, an opposite expression pattern for the two *CTSW* isoforms was observed. The difference between the two isoforms, ENSMFAG000000022903 (E::22903) and ENSMFAG000000022907 (E::22907), was the sequence completeness of the protein domain region, Peptidase_C1. E::23907 had a truncated version of the domain sequence, and its expression pattern contrasted with that at the gene level, whereas E::22903 possessed the complete domain (Supplementary Fig. 2e and Supplementary Fig. 2f). Additionally, we observed both conspicuous gene and transcript levels changed in the expression of *OAS1* gene at stage G3-G4 (Supplementary Fig. 2c). With increased global concern about severity of the COVID-19, this gene has emerged as a promising candidate receiving much recognition^31^. Of the two protein isoforms primarily translated into human OAS1, p46, with the G allele at the acceptor site (rs10774671) of intron 5, is prenylated at the C-terminus of the coding protein whereas p42 has an A allele at the same site, building a different C-terminus with absent prenylation^32,33^. E::49675.1, one of the macaque *OAS1* isoforms has similar transcript sequence equivalent to p46 protein, including the G allele at the human rs10774671 site and CTIL peptide sequence at the C-terminus (Supplementary Fig. 2g). Another isoform, E::49810.1, matched the human OAS1 p44 protein transcript sequence with an absent CTIL C-terminus. In the G3-G4 stage, we found that the expression level of E::49675.1 decreases relative to that of E::49810.1.

Using transcript-level expression data, we investigated alternative splicing (AS) patterns and protein domain changes. IsoformSwitchAnalyzeR analysis revealed that exon skipping (ES), alternative transcription start site (ATSS), and alternative transcription termination site (ATTS) were the most prevalent AS events in all groups. (Supplementary Fig. 3a). In the G3-G4 stage, ATSS and ATTS gain was more frequent than their loss, and the shortening pattern of the open reading frame was dominant, indicating noteworthy patterns in AS events and protein domain changes in older individuals (Supplementary Fig. 3b).

### Significant patterns of immune changes throughout the lifespan of the cynomolgus macaque

**Fig. 2:**
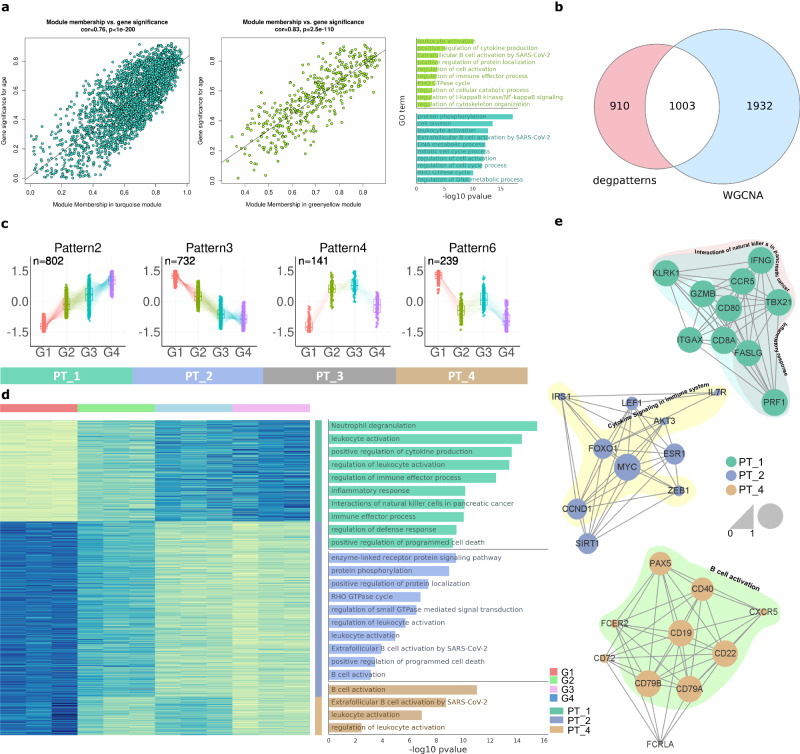
Aging-associated time-series analysis. **a** 2 modules greenyellow and turquoise were identified by WGCNA analysis. Correlation coefficient value of 2 module memberships with gene significance were 0.83 and 0.76 with p values = 2.5e-100 and <1e-200 respectively. GO term of the 2 module were also analyzed with Metascape. **b** Intersection between selected genes from WGCNA (2935) and DEGreport (1913) were 1003. **c** DEGs pattern analysis by degpattern function of R package DEGreport with default setting but cutoff padj < 0.001. Four out of 7 patterns were selected with number of genes > 100. Four patterns were named PT_1, PT_2, PT_3 and PT_4. **d** GO analysis identified 3 patterns (PT_1, PT_2 and PT_4) with the presence of terms. Heatmap of the 3 selected patterns were generated by z-scaled normalization value. **e** Top 10 hub genes of 3 selected patterns with significantly enriched GO terms on the genes. Source data are provided as a Source Data Fig. 2 file.

To pattern the aging-DEGs throughout the lifespan of macaques, we performed a time series analysis at four time using annual blood samples. We used R package’s weighted gene co-expression network anlysis (WGCNA) and DEGreport both coupled with DESeq2. The read count data with low-expression genes removed and normalized using DESeq2 (LRT: likelihood ratio test) were used for WGCNA. Forty-six modules were identified, and MEgreenyellow and MEturquoise were defined as the most positively and negatively correlated modules, respectively, against clinical trait aging (correlation of MEgreenyellow = 0.80, *P* = 2e-06; correlation of MEturquoise = 0.83, *P* = 4e-07) (Source Data). The correlation coefficients of the membership of the two modules with gene significance (GS) were 0.83 and 0.76, suggesting that the two modules were relatively well constructed (MEgreenyellow *P* = 2.5e-110; MEturquoise *P* < 1e-200) (Fig. 2a). GO analysis showed that immune-related terms were significantly enriched on both modules and several terms such as “protein phosphorylation,” “protein ubiquitination,” and “regulation of DNA metabolic process” were enriched only on the MEturquoise module (Fig. 2a). In addition to WGCNA, the DEGreport R package was used for DEG pattern analysis. Read count data normalized by DESeq2 (LRT) were processed in the clustering step using the degPattern function of DEGreport. Seven patterns were identified, and immune-related terms were highly enriched for GO analysis. Intersection analysis of the seven DEG patterns and the pairwise DEGs revealed that 116 of Pattern 2 DEGs were significantly up regulated at the G1-G2 stage (Supplementary Fig. 4a). Of the seven patterns, Patterns 2 and 4 were upregulated, and Patterns 3 and 6 were downregulated throughout aging. We subsequently identified 1003 DEGs between the two selected modules from WGCNA and the four selected patterns from the DEGreport analysis (Fig. 2b). The DEGs from the four patterns were designated new names as PT_1(Pattern 2), PT_2(Pattern 3), PT_3(Pattern 4), and PT_4(Pattern 6), and were used in subsequent steps (Fig. 2c).

GO analysis of the four patterns yielded GO terms for only three patterns: PT1, PT2, and PT4. These findings indicated that immune related terms are associated with aging with the term “leukocyte activation” highly enriched in all three patterns (Fig. 2d). Specifically, “Neutrophil degranulation” and “positivity regulation of cytokine production” were particularly enriched in the Reactome pathway and GO terms in PT_1. The GO terms “enzyme-linked receptor protein signaling pathway” and “protein phosphorylation” were enriched in PT_2. In PT_4, “B cell activation” and “Extrafollicular B cell activation by SARS-CoV-2” were strongly enriched GO terms and WikiPathways (Supplementary Fig. 4b). Additional GO enrichment analyses were performed using the Metascape web tool. For PT_1, terms including natural killer cells, effector T cells and CD8 + T cells were enriched, which was completely different from PT_2 and PT_4. For PT_2, the terms related to naïve T cells were uniquely enriched, various transcription factor targets were enriched compared with PT_1 and PT_4. The enriched terms for PT_4 were similar to those of PT_2; however the B cell-related terms were more enriched (Supplementary Fig. 4d). According to the DisGeNET results, PT_1 genes were associated with various diseases, such as tumors, infections and immunosuppression whereas PT_2 and PT_4 genes were significantly associated with blood tumor such as lymphoma and leukemia.

To obtain a relevant set of DEGs across the lifespan, hub genes of the three patterns were identified using the MCC algorithms of *CytoHubba*. The top 10 genes for each pattern are described in Fig. 2e. Most of the top 10 genes in PT_1 (*CD8A, GZMB, PRF1, TBX21, IFNG, CD80, CCR5, KLRK1, FASLG, ITGAX*) were characterized by the GO term “Interactions of natural killer.” All genes associated with the GO term “Immune effector process,” were related to the innate effector process. The genes in PT_2 (*MYC, ESR1, CCND1, SIRT1, FOXO1, LEF1, ZEB1, IRS1, AKT3, IL7R*) were involved in the “Cytokine Signaling in Immune System,” and the genes in PT_4 (*CD79B, CD79A, CD19, CD22, CD40, PAX5, CD72, FCER2, CXCR5, FCRLA*) were usually marker genes for B cells^21^ (Fig. 2e). In a specific GO analysis of the top 20 hub genes in each pattern, cancer-related terms were highly enriched in PT_1 and PT_2. In contrast, PT_4 remained strongly correlated with terms related to B cell processes (Supplementary Fig. 4e). Therefore, from both GO analysis and hub gene identification, we recognized that the genes of PT_1 were closely related to innate immunity, whereas the genes in PT_2 and PT_4 were associated with adaptive and humoral immunity.

### Specific immune cell composition changes during aging

**Fig. 3:**
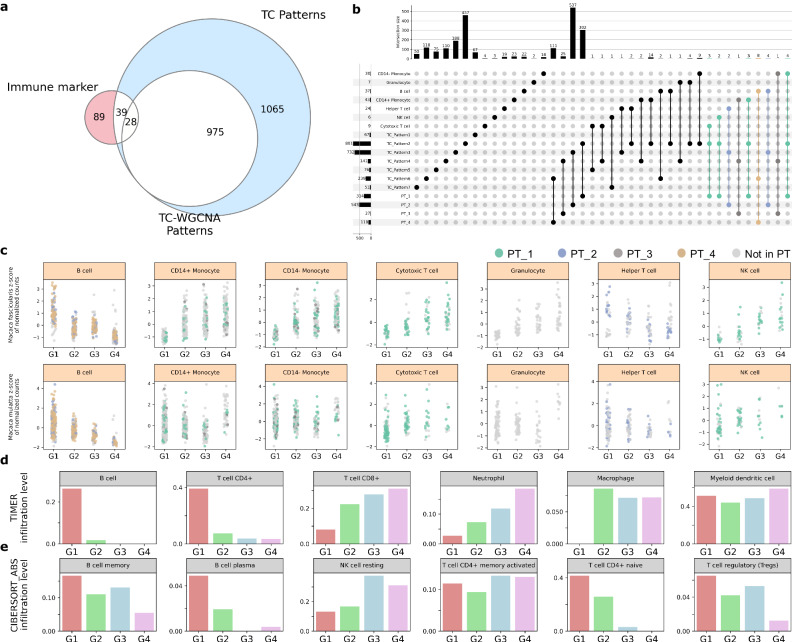
Age-associated immune landscape. **a** Twenty-eight immune marker genes were identified with our selected genes of the 4 patterns. Immune marker genes were generated from *Macaca mulatta* single cell sequencing by previous study. TC-WGCNA patterns indicates genes of selected 4 patterns. TC patterns represents genes of all 7 patterns from time-series analysis. **b** Upset plot of immune marker genes, 7 DEG patterns genes and 4 selected patterns. Colored dots are detailed information of 28 immune marker genes at a. **c** Scatter plots of immune marker gene patterns while aging for *Macaca fasciculairs* from our analyzed data and *Macaca mulatta* from Cayo Santiago population. Colored dots are indication of genes in each 4 selected patterns. Z-scaled normalized value was analyzed in the patternization. Immune type cells shown here are B cell, CD14 + -monocyte, Cytotoxic T cell, Granulocyte, Helper T cell and NK cell. **d**, **e** Immune composition level was examined with TIMER 2.0. TPM values were input at the web version. The result excel data was visualized with customized python code. d is the result from TIMER algorithm and e is from CIBERSORT_ABS algorithm for more specific immune cell status. B cell, CD4 + T cell, CD8 + T cell, Neutrophil, Macrophage and Myeloid dentritic cell were shown. Naïve, regulatory and memory types of T cell and B cell are shown. Source data are provided as a Source Data Fig. 3 file.

Given that specific DEG patterns are related to the immune response during aging, further analysis of the immune cell components in each aging group was performed. First, we determined the number of immune marker genes associated with the identified DEG patterns. The seven DEG patterns from the first time course analysis contained 67 immune marker genes, whereas the four WGCNA-filtered patterns contained 28 immune marker genes (Fig. 3a). Genes with upregulated patterns, such as PT_1, were composed of several immune marker genes related to NK cells, monocytes and cytotoxic T cells. Alternatively, most genes in the downregulated patterns, such as PT_2 and PT_4, were immune marker genes of B cells and helper T cells (Fig. 3b). To investigate the gene patterns of *Macaca mulatta*, we downloaded another aging-associated expression data of *Macaca mulatta* from Cayo Santiago population as these immune marker genes were from *Macaca mulatta* (Methods). Figure 3c shows the patterns of both *Macaca fascicularis* and *Macaca mulatta* using the z-score-scaled median ratio values from DESeq2(LRT) normalization. Immune marker genes of monocytes, NK cells and cytotoxic T cells were upregulated, whereas those of B cells were downregulated (Fig. 3c). Additionally, the seven immune marker genes of B cells were contained, especially in the top 20 hub genes of PT_4, suggesting a strong relationship between B cell decline and aging in macaques (Figs. 2e, 3b, Supplementary Fig. 4c). In helper T cells, genes PT_1 and PT_2 co-existed, but the decreasing pattern was dominant. In the cells, the decreased immune marker genes included *LEF1*, *IL7R*, and *RPS28*, whereas the increased immune marker genes were *JUNB* and *FOS* (Source Data Fig. 3). The activator protein 1 (AP-1), a dimeric transcription factor of Jun and Fos, promotes inflammation in mice as an aging signature^34–36^. In our data, the increased expression of JUNB and FOS, coupled with GO analysis, suggests an association with inflammmaging.

To investigate how the genes of WGCNA-filtered patterns work in the human immune environment, TIMER 2.0 was used for human immune composition estimation. The TPM-normalized value was used as an input, and TIMER 2.0 automatically detected cancer type of DLBC (Diffuse Large B-cell Lymphoma). The results showed that the levels of B cells and CD4 + T cells were downregulated, whereas those of CD8 + T cells and neutrophils increased with age (Fig. 3d). As CD4 + T cells and CD8 + T cells denote helper T cells and cytotoxic T cells, respectively, the results of TIMER in humans corresponded to the immune marker gene patterns of macaque monkey-based DEG patterns. Further results from CIBERSORT_ABS, a built-in algorithm of TIMER 2.0, were generated for more detailed subsets of cell signatures. We found that the numbers of plasma B cells, memory B cells, CD4 + T cells and regulatory T cells decreased with age (Fig. 3e and Supplementary Fig. 5a). Collectively, immune cell analysis demonstrated that cells associated with the adaptive immune system, such as B cells and helper T cells, decease, whereas cells of the innate immune system such as macrophages, neutrophils and NK cells, thrive with age. Notably, cytotoxic T cells increased in a manner similar to that of innate immune cells. This phenomenon appears reasonable based on a previous study that linked the activation of cytotoxic T cells with NK cells against tumors^37,38^. Additionally, regulatory T cells, which are crucial regulators of immune tolerance, declined in response to the deregulation of their core gene *FOXP3*^39^ (Fig. 3e and Supplementary Fig. 5b). The scoring analysis of associated GO terms also showed similar results (Supplementary Fig. 6a, b). Specifically, the scores of the GO terms ‘germinal center B cell differentiation’ and ‘immunoglobulin V(D)J recombination’ associated with antibody diversity gradually declined with aging (Supplementary Fig. 6c, d). Principal component analysis (PCA) of these 20 hub genes correctly separated the samples according to the four age groups (Supplementary Fig. 7a).

### Hub genes reflect age-dependent immunity of human disease

**Fig. 4:**
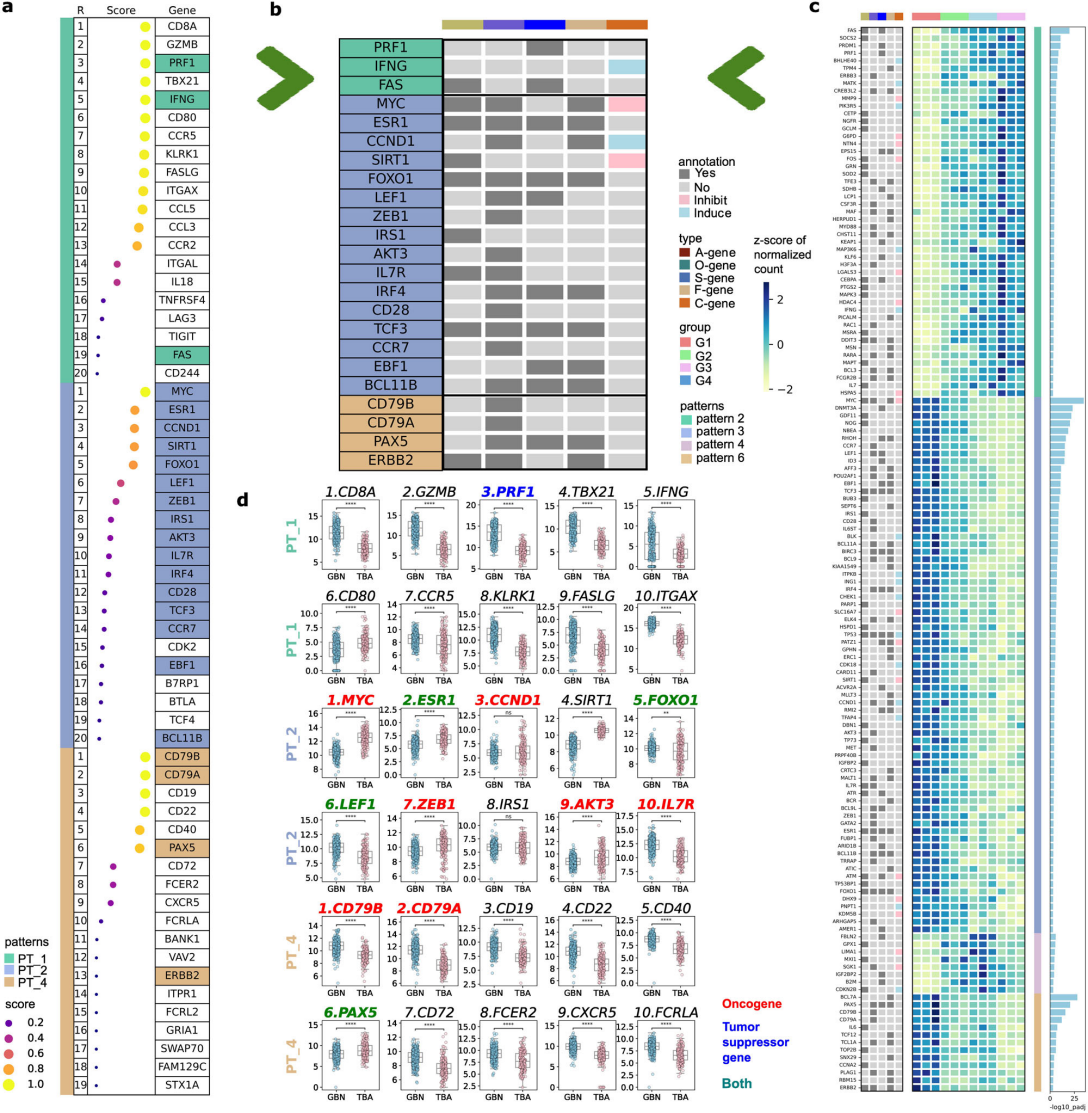
Aging-associated cancer risk of selected hub genes. **a** Top 20 hub genes of 3 selected patterns (PT_1, PT_2 and PT_4). The 1^st^ to 4^th^ column indicate the hub gene rank, score, annotated result from pairwise analysis and the gene symbol. **b** 3 pattern hub genes that have annotated information from the 2 public database. **c** Annotated genes from 4 patterns of time-series analysis. Heatmap visualized with z-scaled normalized value of time-series analysis. Inverse log p value of the analysis are visualized with bar plot. A-gene: aging genes, O-gene: oncogene, S-gene: tumor suppressor gene, F-gene: fusion gene, C-gene: cell-age gene. A- ang C-gene information were from GenAge database and O-, S- and F-gene are from Cancer Census database. Inverse log p value of adjoining pairwise DEG analysis are visualized with up DEG colored red and down DEG colored blue. **d** TCGA(Acute Myeloid Leukemia)-GTEx(normal) gene expression data comparison of top 10 hub genes. Significant difference was calculated by two-tailed Mann-Whitney test with bonferroni correction. ns: 5.00e-02 < p <= 1.00e + 00, *: 1.00e-02 < p <= 5.00e-02, **: 1.00e-03 < p <= 1.00e-02, ***: 1.00e-04 < p <= 1.00e-03, ****: p <= 1.00e-04. Source data are provided as a Source Data Fig. 4 file.

The three patterns of monkey-based DEGs led to speculation regarding their application in human aging research. We subsequently assessed the number of genes within the patterns that were involved in the cancer process, a key risk factor for which has been reported as aging^40^. The top 20 genes from the three patterns were listed and cross-checked with gene lists from GenAge and COSMIC databases (Fig. 4a, c). Notably, nine hub genes were identified as annotated aging genes and 20 hub genes were identified as specific tumor regulation groups. Among these 20 hub genes, 17 of PT_2 and PT_4 had oncogenic features whereas two of PT_1 had tumor-suppressor characteristics (Fig. 4b). When considering all the analyzed hub genes, 22 were classified as oncogenic and 11 as tumor suppressors among the annotated 41 hub genes, and 8 had both features (Supplementary Fig. 7b). Notably, the majority of PT_2 hub genes had oncogenic characteristics, indicating that downregulated gene patterns strongly inhibit tumor growth during aging. The two types of patterns mentioned earlier showed similar division to the tumor regulation features with strong correlation of PT_2 with oncogenic features. To further investigate this, we examined their gene expression in human cancer data (Fig. 4b and Supplementary Fig. 7b). We compared hub gene expression in acute myeloid leukemia(AML) samples to that in normal samples. The top ten hub genes of PT_2, which have oncogenic-oriented characteristics, showed increased expression in AML, in contrast to their aging patterns. However, the hub gene expression of PT_4 decreased in AML cells, similar to the aging pattern (Fig. 4d).

As immune abnormalities cause various diseases, we further determined whether hub genes can be applied to human disease data^41^. We downloaded the human transcriptome read counts from the GTEx portal and selected human male blood samples because our macaque data consisted of blood samples from male individuals. Among the four death classes of the samples, those with less than 1 h of the terminal phase before death were grouped into the normal category while the others were categorized into illness class 1 and illness class2, which are abnormal depending on the duration of the terminal phase. For normal samples, marginal upregulated patterns for PT_1, and PT_2, and PT_4 showed downregulation with age, similar to those of macaque samples (Supplementary Fig. 8a, b). However, for the Abnormal samples, the illness class 1 pattern began to deviate from the normal pattern and the illness class 2 pattern was almost destroyed. These results indicated that long-term exposure to the symptoms of the disease can dysregulate the immunological pattern, leading to death.

### Age estimation of selected hub genes is consistent with chronological age

**Fig. 5:**
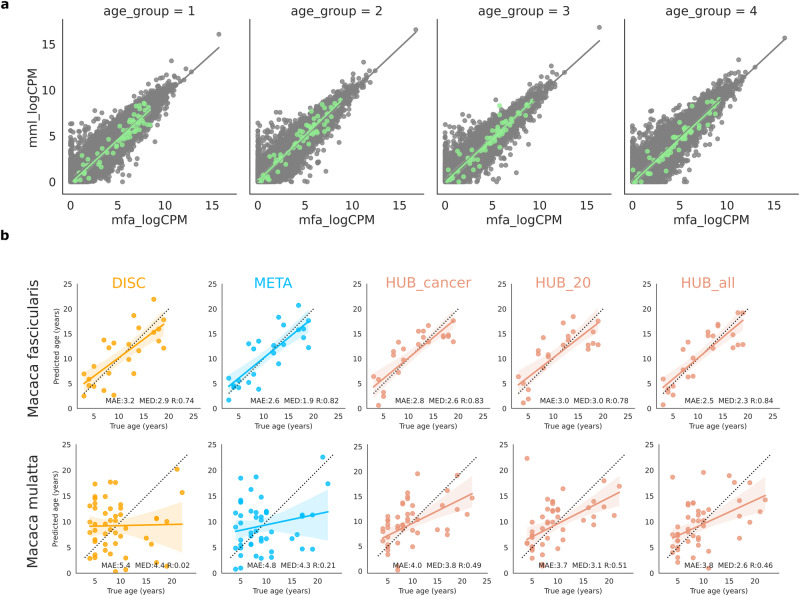
Transcriptomic age prediction. **a** Scatter plot depicting similarity of gene expression for our data of *Macaca fascicularis* versus Cayo Santiago population of *Macaca mulatta*. Log_2_-transformed counts per million (LogCPM) of both macaques gene expression values were used. Black and green dots represent all overlapping genes and all hub genes respectively. **b** Transcriptomic age prediction of both macaques. LogCPM were used for expression values of the prediction. Transcriptomic age was estimated with the method introduced by previous studies and compared with the true age. The comparison was evaluated with the metrics of mean absolute error (MAE), median absolute error (MED), Pearson’s correlation (R). Source data are provided as a Source Data Fig. 5 file.

Next, we estimated the age using the selected hub genes. As age prediction using multi-omics data, especially those of the methylome and transcriptome, is currently a vibrant research area, we examined the influence of age-dependent hub genes on this estimation^42^. We first confirmed that the 60 hub genes from *Macaca fascicularis* were expressed in *Macaca mulatta* from Cayo Santiago population in a similar manner (Fig. 5a). We subsequently applied the previously published prediction algorithm by Peters et al. to both species (Source Data Fig. 5). Calculation using our hub genes showed similar or less error rates than those using previously defined values in both species (*Macaca fascicularis* : mean absolute error of 2.8, 3.0, and 2.5 years for hub genes). Moreover, higher R^2^ values for true versus predicted age were calculated for *Macaca fascicularis* (R = 0.83, 0.78, and 0.84 for hub genes) (Fig. 5b). We extended the prediction to human aging metadata from Mendeley data which is divided into two groups: healthy and unhealthy. In the healthy data of the 222 blood samples, according to the resulting mean error rates and R^2^ values, prediction using hub genes marginally outperformed the application of previous methods. Conversely, unhealthy samples showed relatively poor performance even in our hub genes (Supplementary Fig. 8c). Thus, these results demonstrated that our analyzed hub genes in this study similarly or a bit better represent aging phenomenon in both humans and macaques than the one in previous studies.

### Transposable elements (TEs) analysis also shows age-related pattern

Next, we investigated whether there were specific age-related phenomena in mobile elements of cynomolgus macaques. TEs that constitute nearly half of the primate genome have recently received attention from industries as promising regulatory factor^43^. As TE transcription occurs during the retrotransposition cycle and its expression is diverse depending on certain genomic states and tissues, measuring TE transcripts can serve as a genomic indicator that elucidates genome instability^44–46^. In this study, we measured TE expression levels using TE read counts from the same RNA-seq data used for the DEG analysis. The total expression levels of the primary four types of TE family members (LINE, SINE, LTR, and DNA transposons) gradually increased with age, as mentioned in previous studies^47^ (Supplementary Fig. 9c). Differentially expressed TEs (DETEs) analysis was also conducted using the time-series method, and six patterns including two up and down patterns, were identified (Supplementary Fig. 9d, e). In Pattern 4, the upregulated pattern was more abundant than the downregulated Pattern 2, especially for the SINE family (Supplementary Fig. 9c and Supplementary Fig. 9a). Among them, *MIRb* was significantly more than double the amount observed in Pattern 4 (Supplementary Fig. 9b). From a whole-genome perspective, chromosome 19 (Chr19), high-gene-density part of human genome, exhibited a markedly increased number of DETEs despite its relatively short genomic size^48^ (Supplementary Fig. 9f). The number of TEs on Chr19 was marginally higher than that on any other genomic chromosomes, however, the number of DETEs was considerably higher on this chromosome.

## Discussion

Although the latest aging research has been conducted in various fields with large volumes of data, the aging phenomenon itself remains obscure. To obtain details of the plausible biological mechanism of aging, we conducted and applied various correlation analyses and research tools to the whole transcriptome data of laboratory monkeys. In contrast to traditional aging research that typically compares young and old groups, we established four age groups and identified both the features and life-long patterns of aging. There is close link between the aging process and the immune system^12,49^, and this was also observed in this study. Furthermore, we characterized the immunologically altered patterns of aging, identified the hub genes of the patterns, and determined then the reliability and applicability of the pattern groups via a statistical comparison of our data with the equivalent data of *Macaca mulatta* and humans.

Among two major patterns identified in this aging study, PT_1 was primarily associated with innate immunity, as indicated by the presence of numerous marker genes for innate immune cells, including macrophages, NK cells, and neutrophils. Considering that innate immunity functions as the first line of defense against pathogen infection, upregulated PT_1 appeared to enhance initial immune activity during aging. This concept is particularly relevant when we consider G1-G2 stage of the study, as the equivalent stage from infancy to adolescence represents the period of most healthy innate immune response throughout life^50^. However, we observed that antiviral genes, such as ISG15 and IFIT family genes were downregulated at the G2-G3 stage, suggesting a gradual loss of antiviral activity in older individuals (Source Data)^51,52^. Even more concerning during aging is that long-term stimulation of innate immunity, as observed in the upregulation of PT_1, can lead to chronic inflammation, a crucial risk factor for age-related diseases^12^. We also observed an increased expression of *JUNB* and *FOS*, which are members of the AP-1 family that promote inflammation. Simultaneously, we observed adaptive immunity in which CD8 + T cells thrived. This was coupled with the upregulation of *PRF1* (Perforin-1) and *GZMB* (Granzyme B) genes in NK cells. Groh, J et al. showed that axonal degeneration in the central nervous system of aged mice is caused by cytotoxic CD8 + T cell accumulation, and this can be exacerbated by systemic inflammation^53,54^. Conversely, PT_2 downregulation was primarily associated with CD4 + T and B cells. We observed a decline in naïve CD4 + T cells and regulatory T cells, and an increase in memory T cells^52^. In addition, the immune marker genes of B cells were closely associated with downregulated PT_4 and decreased plasma cell levels. The pathways related to cancer such as “PI3K/AKT Signaling in Cancer”, were enriched with the GO term “leukocyte differentiation” for the top 20 genes of PT_2. Collectively, in conjunction with PT_2 and 4, we observed that adaptive immunity diminishes, prompting immunosenescence. This reduction in T cell proliferation, B cell lineage diversity, and antibody affinity can be detrimental to the older immunity^55^. Simultaneously, the dysregulation of diminishing oncogenic PT_2 genes could be a causal factor to cancer development.

**Fig. 6:**
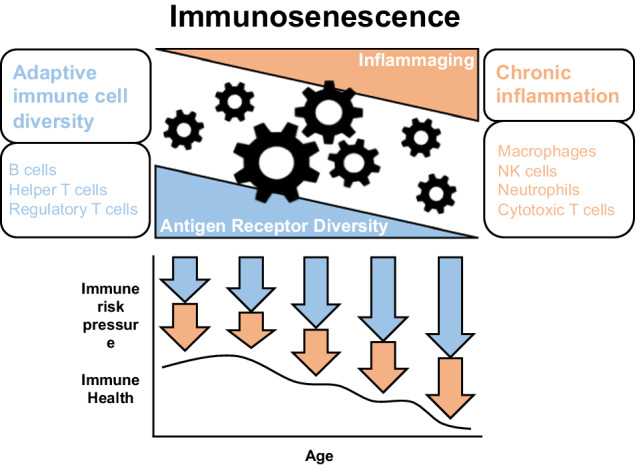
Overview of the study. Up-regulated expression pattern of the certain innate immune cells in early child stage works well stopping the invasion of the viruses but it chronologically dampens the total immune power of the old individuals. The down-regulated pattern of certain adaptive immune cells reduces the diversity of the cells during aging. The cogwheels represent other interconnected features related to aging. All those patterns and features in the old individuals operate as immunological risk factors to aging.

With the identified up and down patterns, we were able to characterize the age-associated immunological landscape described in Fig. 6. Low-grade infectious pathogens chronically stimulate the innate immune system of host individuals without causing severe symptoms over a life-long period, activating a gradual increase in inflammation. Although the innate defense mechanism was observed to develop during the early stages of life, our data showed the loss of antiviral mechanisms after adolescence, potentially exacerbating chronic inflammation. In addition, the adaptive immune capacity progressively diminishes during aging. The gradual loss of diversity of naïve T and B cells are considered the cause of another part of immunosenescence. Collectively, these primary patterns of immunosenescence seem to exacerbate body frailty. In addition, we revealed several minor patterns that deviated from the main trajectories. For example, *CTSW* and *OAS1* genes, as observed in the pairwise analysis, displayed fluctuating patterns. According to a study by Edinger et al., influenza A virus accumulation is proportional to the expression level of *CTSW*^56^, and the reduced expression pattern of the gene in the study during the G1-G2 stage appears to be an essential phenomenon contributing to the resistance to virus infection during adolescence stage. Peptidase_C1 domain of the gene seems to play a critical role in viral growth during puberty. In the case OAS1, Wickenhagen et al. insisted that the prenylated C-terminus, with its peptide sequence CTIL (CAAX motif) transports the OAS1 protein to the endomembrane system, leading to efficient antiviral activity^31^. Therefore, the decreased expression level of E::49675.1 containing CTIL peptide sequence in the G3-G4 stage may be a possible cause of increased susceptibility to viral infection in older individuals. Several genes have different tumor regulation characteristics that are different from the main characteristic. In PT_2 and PT_4, most of the downregulated hub genes were oncogenic; however, several genes were tumor suppressors. Conversely, in PT_1, the opposite pattern was observed. These results demonstrated that upregulated oncogenes and downregulated tumor suppressor genes during aging can be causal factors of diseases in older individuals. Collectively, we found that whole transcriptomic immune patterns can affect the aging process, even if the pattern was beneficial at an early stage. When we explored the GTEx and Mendeley data, the hub genes also partially explained the healthy state of human aging. Therefore, our hub genes may be useful as powerful candidates for aging research in both human and non-human primates. Additionally, analysis of the TE transcript revealed several age-associated features; however these results require further examination. As our data were limited to male individuals and sex-dimorphism in immune aging has been described at previous studies^57^, our study also warrants further investigation in female individuals.

The importance of immune aging has grown rapidly in recent aging societies with extended life expectancies leading to various health problem. In this study, we found that the immune response evolved against pathogen invasion, gradually affecting frailty, and additional dysfunction or dysregulation accelerated this process. Our results showed that several immune pattern changes constitute a specific immune status during aging. Defining a favorable immune status at a life stage will greatly benefit recent technologies in cancer therapy and vaccination; however, such technologies are considerably affected by age-related issues. Therefore, our study provides new transcriptomic insights into the age-dependent immune status. Future studies should include comparison with other omics data, particularly epigenetic data, to provide more reliable results in the field of aging.

## Methods

### Study samples and ethics

Crab-eating monkeys (*Macaca fascicularis*) were provided by the National Primate Research Center of the Korea Research Institute of Bioscience and Biotechnology (KRIBB). Eight healthy and specific pathogen-free monkeys were selected, and sampling was conducted over 3 consecutive years. Whole blood was drawn annually from the femoral region of each participant. The monkeys were anesthetized with a ketamine (5 mg/kg) injection before sampling. All monkeys underwent a complete physical assessment. For viral (simian T-cell lymphotropic/leukemia virus-1 and -2, simian immunodeficiency virus, simian retrovirus-1, -2, -5, and simian virus 40), bacterial (*Mycobacterium tuberculosis* (TB), *Shigella* spp., *Salmonella* spp., and pathogenic *E. coli*), and parasite examinations were conducted, and all results were negative. The monkeys were housed in an indoor room with a constant temperature of 24 ± 2 °C, 50 ± 5% relative humidity, 100% fresh air at ≥12 room changes/h, and a 12 h light:dark cycle. The monkeys were provide with identical diets and food conditions. The attending veterinarian performed annual health monitoring according to the recommendations of the Weatherall Report (https://royalsociety.org/topics-policy/publications/2006/weatherall-report). All animal procedures were performed in accordance with the Guidelines of the Institutional Animal Care and Use Committee of the KRIBB (approval no. KRIBB-AEC-18087, KRIBB-AEC-19046, KRIBB-AEC-20217).

### RNA extraction and high-throughput sequencing of RNA

Total RNA was extracted from the blood samples using a PAXgene Blood RNA Kit (Qiagen, GmbH, Germany). mRNAs were isolated from 1 µg of total extracted RNA. Paired-end sequencing libraries (151 bp) were prepared using TruSeq Stranded mRNA Sample Preparation Kit (Illumina, CA, USA). Library quality was evaluated using the Agilent 2100 BioAnalyzer (Santa Clara, CA, USA). A KAPA Library Quantification Kit (Kapa Biosystems, MA, USA) was used to quantify the libraries. The constructed libraries were sequenced using an Illumina NovaSeq 6000 (Illumina).

### RNA data preprocessing

Raw sequencing data was processed to filter out dirty reads using the following criteria, i) reads with > 10% ‘N’ bases; ii) reads with a low-quality threshold of 40% with a Q ≤ 20 bases; and iii) reads with an average quality score < 20. RNA sequencing and raw data filtering were performed by Theragen Bio (Seongnam, South Korea). As this study covered the whole transcriptomic data, including mRNA and TE transcripts, SQuIRE^58^ (https://github.com/wyang17/SQuIRE) was used for read alignment and quantification. The filtered TE transcripts were mapped to the reference genome (*Macaca fascicularis*) (https://hgdownload.soe.ucsc.edu/goldenPath/macFas5/bigZips/macFas5.fa.gz) using the SQuIRE Map function. Owing to different mapping conditions, filtered mRNA reads were mapped to the same genome using STAR v.2.7.9a. The gtf file was downloaded from the same UCSC database (https://hgdownload.soe.ucsc.edu/goldenPath/macFas5/bigZips/genes/ macFas5.ncbiRefSeq.gtf). Expression estimation from both mapped BAM files was performed using the SQuIRE Count function. The count value of each sample was used for downstream analysis of DEGs.

### Gene expression analysis

The batch effects of the count values were removed using ComBat-Seq tools in R^59^. Subsequent normalization and significant DEG detection was performed using DESeq2 (version 1.36.0)^60^. The Wald test was run for adjoining group pairwise analysis of the two age groups, and the LRT was run for time-series analysis of the four age groups. Normalized counts were z-score scaled for visualization in an expression heatmap. In the time-series analysis, specific patterns were defined using the degpattern function of R package’s DEGreport (version 1.28.0) (http://lpantano.github.io/DEGreport). For stringent cut-offs, padj < 0.001 was used for the analysis (https://hbctraining.github.io/DGE_workshop/lessons/08_DGE_LRT.html). These processes identified seven specific patterns in the transcriptomes of the aging monkeys. The four patterns were selected based on the condition that the number of genes in each pattern was >100. WGCNA^61^ was performed separately with default settings, and employing a soft threshold power of 7 and an R^2^ cut-off of 0.8. Vst-normalized data were used as inputs for WGCNA in accordance with the tutorial recommendations (https://horvath.genetics.ucla.edu/html/CoexpressionNetwork/Rpackages/WGCNA/Tutorials). Two modules (MEgreenyellow and MEturquoise) significantly associated with aging were selected (Source Data). We identified the genes shared by both the four patterns and two modules and visualized them using patterned images. Finally, three patterns (PT_1, PT_2 and PT_4) that identified specific GO terms were selected for analysis. For counts per million (CPM) values, the input for the Python package bioinfokit (version 2.0.6) (10.5281/zenodo.3698145) consisted of batch-corrected counts. Log_2-_transformed counts per million (logCPM) values were used for expression data comparison between humans and *Macaca mulatta*. To analyze TE expression, TE count values were interpolated to mitigate substantial data loss. This was necessary owing to the relatively lower expression values of TE than gene expression, which resulted in numerous omitted values among six samples in each age group. Subsequently, the same downstream method was used for the TE count.

### Differential transcript usage (DTU) analysis

The transcript levels of differential expression were investigated to confirm the usage of transcripts during aging. Raw RNA sequencing data used for RNA data preprocessing were aligned and quantified with the reference genome and corresponding annotation data of *Macaca fascicularis* downloaded from Ensembl version 102 using Kallisto (version 0.46.1)^62^. The DTU analysis was performed using the R package’s IsoformSwitchAnalyzeR (version 1.12.0)^63^. Isoform switches were predicted using DEXseq, GenomeObject of BSgenome.Mfascicularis.NCBI.5.0, and default parameters. External sequence information was annotated using the generated result files from CPC2, Pfam, IUPred2A, and SignalP. Isoforms by default settings were selected for further analysis.

### Gene ontology (GO) analysis

Human orthologous gene symbols from DEGs were determined using R package biomaRt (version 2.25.0). These gene symbols were used as inputs for running Metascape (https://metascape.org) and STRING (https://string-db.org) web tools with default setting^64,65^. The analyzed GOs were visualized using a customized Python code. Furthermore, several GO terms (https://geneontology.org) were scored for detailed characterization. We applied the scoring method devised by a study of Xie X et al.^66^.

### Hub gene identification

The resulting TSV file from STRING was analyzed using Cytoscape (version 3.9.1)^67^ for hub gene identification. The MCC algorithms of the cytohubba plugin were used to obtain the scores of hub genes^68^. The three groups of hub genes were separately selected for further examination: i) 59 genes, which were the top 20 hub genes of each of the three selected patterns (19 genes for PT_4), ii) 20 genes that had oncogenic or tumor suppressor characteristics from the 59 hub genes, iii) 605 genes that were all scored as hub genes from the three patterns.

### Immune composition analysis

Immune cell composition in our data was estimated based on both human and *Macaca* lineages. The TIMER 2.0 web tool^69^ was used for human-based analysis with the TPM-normalized expression value generated by SQuIRE. With our data, TIMER 2.0 automatically detected cancer type of DLBC (Diffuse Large B-cell Lymphoma). For immune composition analysis in the *Macaca* lineage, immune marker genes of *Macaca mulatta* were downloaded from a study by Watowich et al. for *Macaca*-specific analysis^70^. To represent the aging of *Macaca mulatta*, their transcriptome data in the Cayo Santiago population were downloaded^71^. Using *Macaca*-specific immune marker genes, the z-score-scaled normalized expression values of DEGs were evaluated to recognize patterns and specific genes that constitute the specific immune cells for both *Macaca* species. The results of immune cell composition obtained from TIMER and CIBERSORT_ABS^72^ tools within TIMER 2.0 were further analyzed and interpreted.

### TCGA-GTEx data analysis

Public cancer transcriptomic data of the hub genes were analyzed to examine the tumor regulatory features of the aging patterns. The RNA-seq gene expression data file “TcgaTargetGtex_RSEM_Hugo_norm_count.txt” based on TCGA, TARGET, and GTEx data sets and its annotation data file “TcgaTarget GTEX_phenotype.txt” were downloaded from UCSC Xena (http://xena.ucsc.edu/). Normal blood GTEx samples were extracted as controls, and AML samples, which were the only primary blood samples derived from TCGA, were selected as cancer samples. The normalized expression counts of each hub gene in control cancer samples were compared. Statistical significance was calculated using the Mann–Whitney–Wilcoxon test.

### Pattern examination in the human transcriptome

Human transcriptome read count data with annotation information were downloaded from the GTEx portal (GTEx Analysis V8)^73^. Among the 17,382 samples, blood samples from males (*n* = 929) were sorted to match with the corresponding blood samples of male monkeys. The GTEx samples were classified into four groups based on the duration of exposure to the cause of death. Samples from classes 1 and 2 belonged to patient who died in accidents or other unexpected natural causes less than 1 h before. The samples of classes 3 and 4 were belonged to patient who died owing to illness within 1 h< and <24 h, and >24 h, respectively. In this study, samples of classes 1 and 2 were defined as normal, class 3 as illness_1, and class 4 as illness_2. The additional class 0, which pertains to ventilator cases, was excluded because of a lack of phase information.

### Transcriptomic age estimation

Transcriptomic age prediction for *Macaca fasiscularis*, *Macaca mulatta* (Cayo Santiago rhesus population) and normal and abnormal humans was performed. CPM values were used to estimate the age. The batch effects of our transcriptomic CPM sampling were eliminated using sufficient sampling information. The age prediction approach developed in the study by Peters et al. for humans was applied to this analysis^4^. The transcriptomic age predictors were obtained from Supplementary Data 1 and 5 of that study paper. Age prediction of the hub genes was performed using Equations 12 and 13 from the study. The gene groups, DISC and META, were performed together for comparison. The genes of DISC (11,908 genes) and META (1497 genes) were significantly expressed in 7074 human genome samples and further studies with 7909 more samples respectively from the study of Peters et al.^4^.

Both equations were run using a customized Python code upgraded from the study by Kenneth L et al.^71^. Hub gene symbols were converted to the equivalent human gene symbols using biomaRt. The prediction was evaluated using the metrics of mean absolute error (MAE), median absolute error (MED), and R^2^ according to the study by Fleischer et al.^74^. Data from Mendeley (https://data.mendeley.com/datasets/92rgnswtn8/1) were downloaded for use in the analysis of human aging, and blood samples were selected for subsequent step in which 222 healthy samples and 51 unhealthy samples were used^75^.

### Supplementary information

Supplemental material

Source Data Fig.1

Source Data Fig.2

Source Data Fig.3

Source Data Fig.4

Source Data Fig.5

### Source data

Source data

## Data Availability

RNA-seq data used in this study has been deposited in NCBI as Bioproject #PRJNA1121919 and the Korea Sequence Read Archive (KRA) of Korea Bioinformation Center, Korea Research Institute of Bioscience and Biotechnology (KRA: KAP230570) which is publicly accessible at https://kbds.re.kr/KRA. Other downloaded data used in this study are available on the websites mention in the Methods. Python, R packages, and other software used in this study were obtained from open sources. The source data underlying Figs. 1–5 are provided as Source Data Fig. 1, Source Data Fig. 2, Source Data Fig. 3, Source Data Fig. 4 and Source Data Fig. 5 files respectively. The additional data generated by this study is provided as Source Data file.

## Code availability

All codes designed using these programs are accessible via GitHub at (https://github.com/hmcvz/CynomolgusTranscriptomeAging).
