# Supplementary material for: Transcriptome analysis of cynomolgus macaques throughout their lifespan reveals age-related immune patterns
Source: NPJ Aging. 2024 Jun 20;10(1):30. doi: 10.1038/s41514-024-00158-0 (PMC11189941; doi:10.1038/s41514-024-00158-0)
Supplement: Supplementary file 1 — Supplemental material [file 41514_2024_158_MOESM1_ESM.pdf]

Supplementary Fig. 1

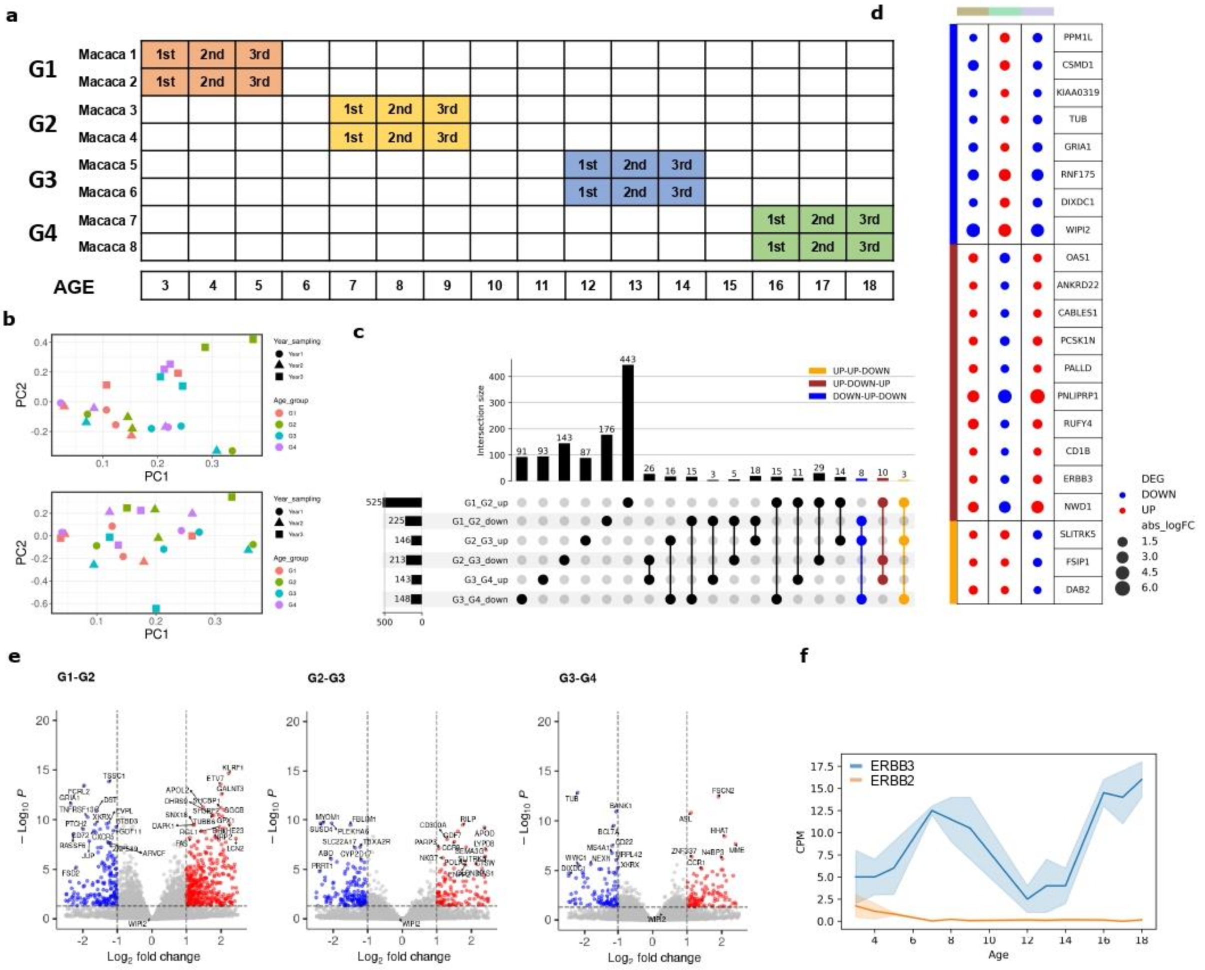

Supplementary Figure 1 : Nearby age group pairwise analysis.

a Three years of blood sampling design **b** PCA results of batch effects correction by R package Combat Seq. upper: PCA result of read count data before batch effects removal. Bottom: PCA result after batch correction. **c** Upset plot for all up and down DEGs of each age stage. Colored dots are involved in all 3 stages. yellow: up-up-down, brown: up-down-up, blue: down-up-down. **d** Detailed information of the genes at Figure S1b which significantly cover all 3 stages. Size of dot represent absolute value of LogFC, and red, blue indicate positive and negative value of LogFC. **e** Volcano plots of 3 adjoining age groups (G1-G2, G2-G3 and G3-

G4) pairwise analysis. Dots represent single genes and x axis is $\log_2$fold change (LogFC) and y axis is inverse log-transformed p-value ($-\log_{10}P$). Blue and red color indicate the genes with p-value < 0.05, LogFC < -1 and p-value < 0.05, LogFC > 1 respectively. **f** Line plot of two genes *ERBB2* and *ERBB3*. The CPM value of each age sample was plotted.

Supplementary Fig. 2

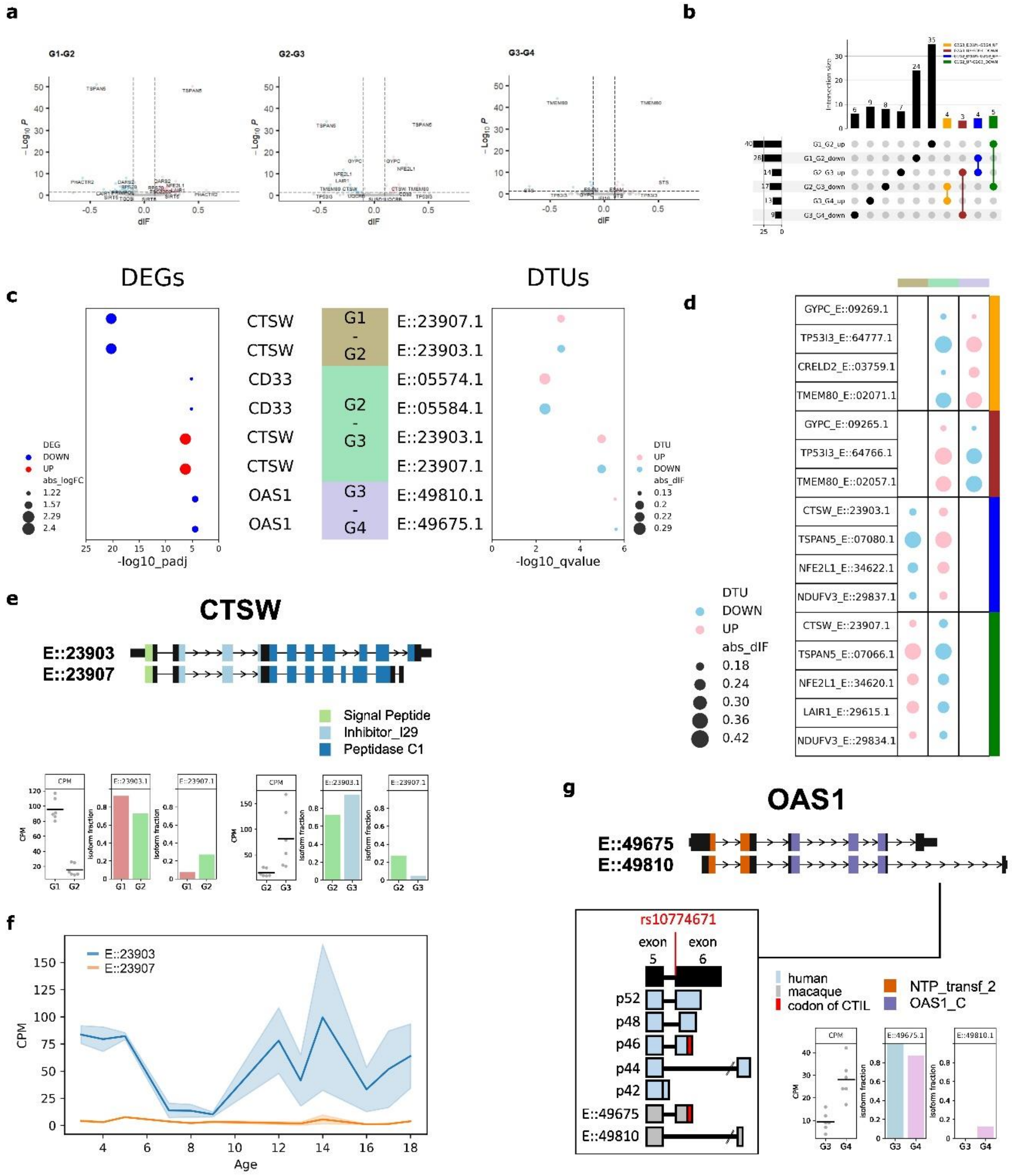

Supplementary Figure 2 : Transcript level adjoining age group pairwise analysis.

a Volcano plots of 3 adjoining age groups (G1-G2, G2-G3 and G3-G4) transcript level pairwise analysis. Dots represent single transcripts and x axis is dIF and y axis is -$\log_{10}$P. Skyblue and

pink color indicate the genes with p-value < 0.05, dIF < -0.1 and p-value < 0.05, dIF > 0.1 respectively. **b** Upset plot for all up and down DEGs of each age stage. Colored dots are involved in all 3 stages. yellow: up-up-down, brown: up-down-up, blue: down-up-down **c** Correlation between significant DEGs and DTUs were examined. Size of dot indicates $\log_2$ fold change on DEGs and dIF on DTUs. Red dot represent positive value meaning increase while blue is negative value meaning decrease. **d** Detailed information of the transcripts at Figure S2b which significantly cover more than 2 stages. Size of dot represent absolute value of differential isoform fractions (dIF), and pink and skyblue indicate positive and negative value of dIF respectively. **e** Isoform switch changes of gene *CTSW* on the stages. Upper panel is transcript structure with differentially colored depending on protein domain. Red, green and blue indicates G1, G2, and G3 age group respectively. **f** Line plot of two transcripts E::23903 and E::23907 of *CTSW* gene. The CPM value of each age sample was plotted. **g** Isoform switch changes of gene *OAS1*. Upper panel is transcript structure differentially colored with each protein domain. Blue and pink indicates G3 and G4 respectively. Sub box shows publicly registered transcripts structure of exon 5 and 6 for human and *Macaca fascicularis.*

Supplementary Fig. 3

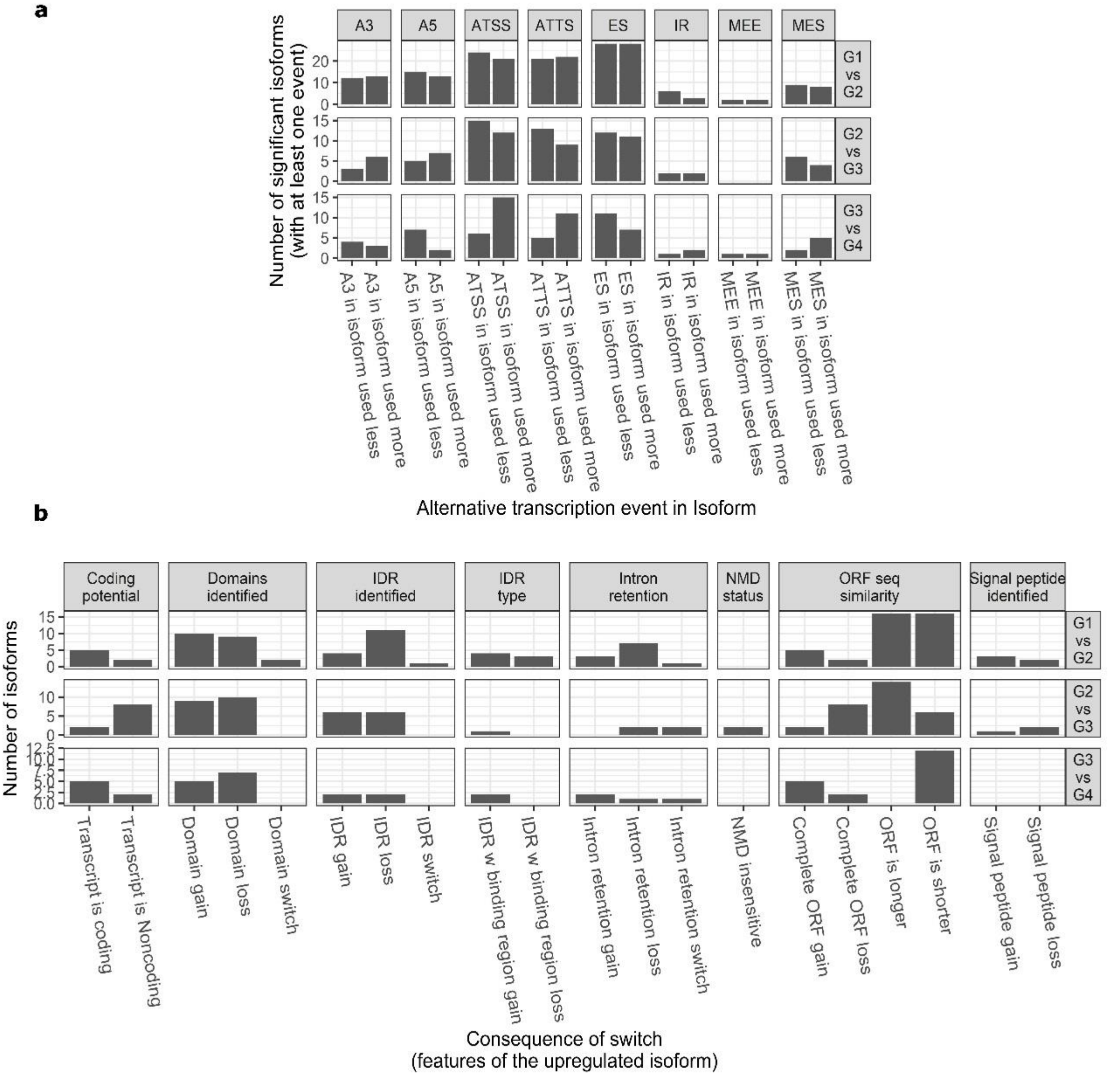

Supplementary Figure 3 : Differential usages of Alternative splicing (AS) event while aging.

a Bar plot of gain or loss of AS event occurrence. Y axis is the number of AS event and x axis is gain or lose count. A3, A5, ATSS, ATTS, ES, IR, MEE and MES indicate alternative 3' acceptor sites, alternative 5' acceptor sites, alternative transcription start sites, alternative transcription termination sites, exon skipping, intron retention, mutually exclusive exons, and

multiple exon skipping respectively. **b** Bar plot of isoform switch details. Y axis is the number of isoform switched and x axis is the certain feature of unregulated isoform. IDR NMD and ORF are the abbreviation of Intrinsically Disordered Regions, Nonsense Mediated Decay and Open Reading Frame respectively.

Supplementary Fig. 4

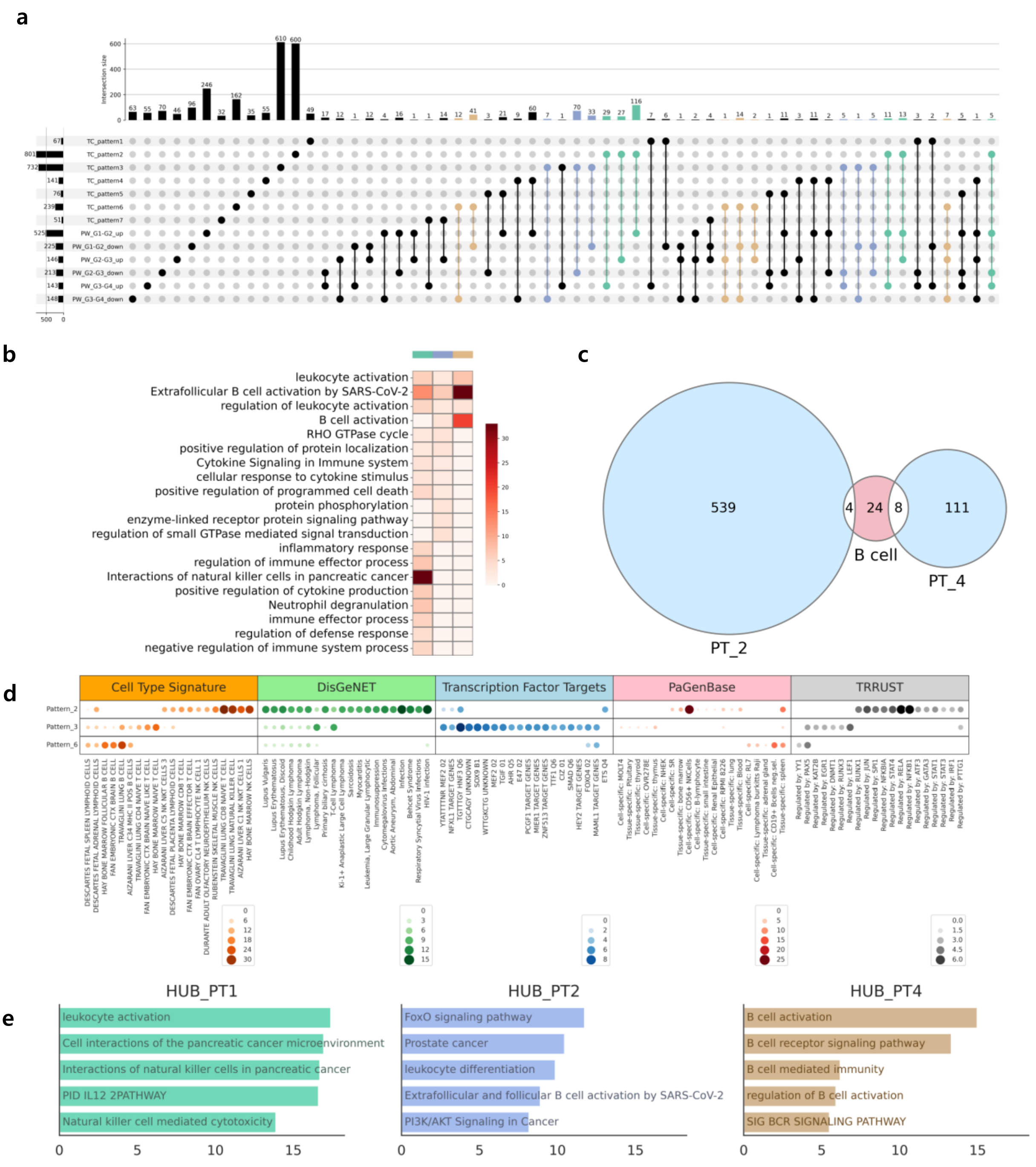

Supplementary Figure 4 : Additional annotation of selected time-series patterns.

a Upset plot for all up and down DEGs with time-series DEGs. Colored dots are all involved with 3 patterns (Pattern 2, Pattern 3 and Pattern 6). **b** Heatmap of GO term from Metascape

analysis. GO enrichment scores of 3 selected pattern (PT_1, PT_2 and PT_4) were visualized. **c** Venn diagram depicting intersectional parts of B cell marker genes (*Macaca mulatta*) with PT_2 and PT_4 which are down regulated patterns of the analysis. **d** Dot plot showing comprehensive gene annotation from several public DB DisGeNET, PaGenBase, TRRUST and etc using Metascape platform. Size of dot represent $-\log_{10}P$ value of each descriptive terms. The row of number is the rank by score of each patterns. **e** GO analysis of top 20 hub genes of each 3 patterns (PT_1, PT_2 and PT_4). Top 5 terms are shown. X-axis represent $-\log_{10}P$ value of each terms.

Supplementary Fig. 5

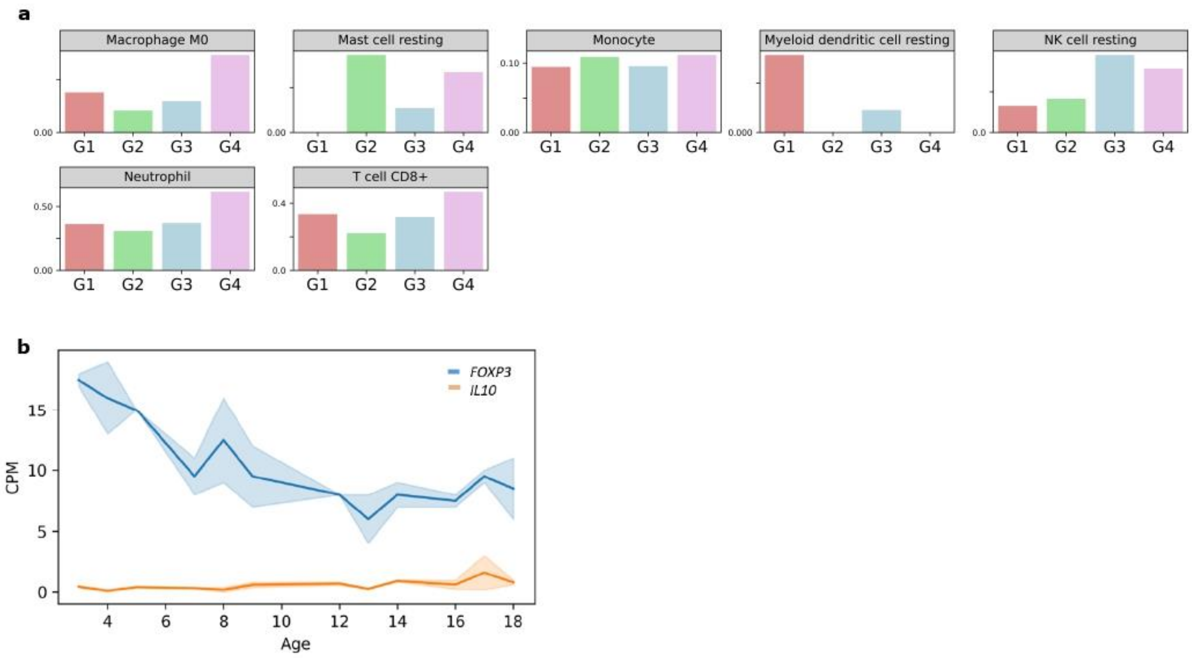

Supplementary Figure 5 : Immune genes.

a CIBERSORT_ABS algorithm for more immune cell status. Machrophage M0, Mast cell resting, Monocyte, Myeloid dendritic cell resting, NK cell resting, Neutrophil and CD8+ T cell were shown. **b** Line plot of two genes FOXP3 and IL10. The CPM value of each age sample was plotted.

Supplementary Fig. 6

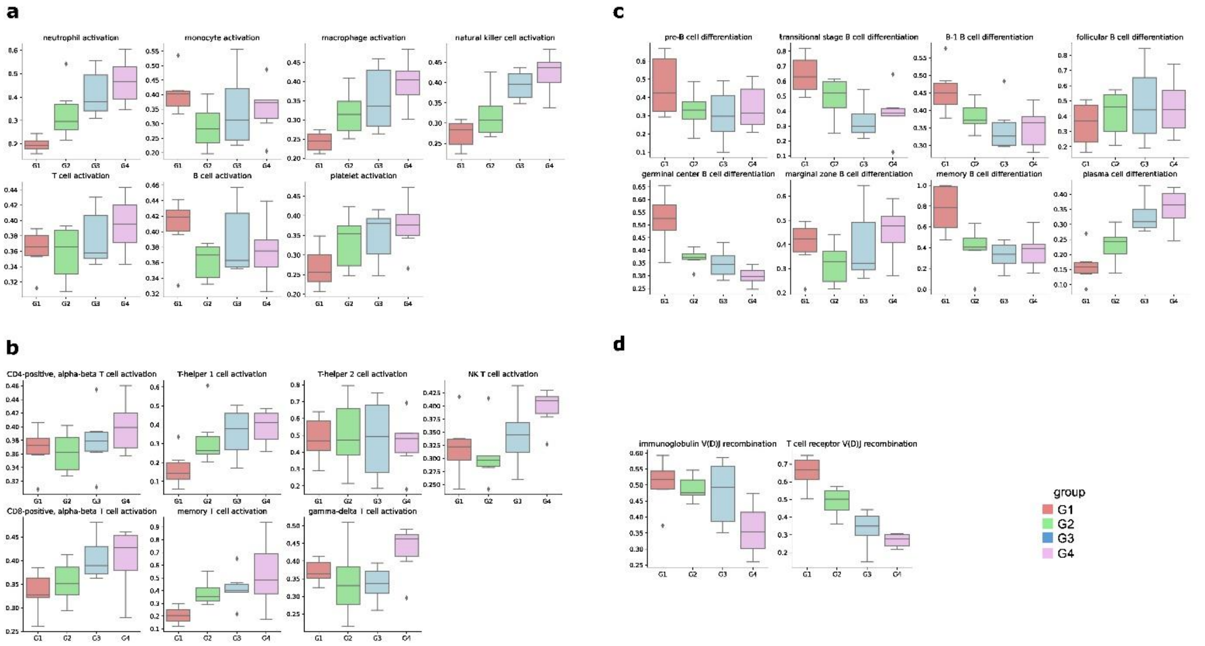

Supplementary Figure 6 : Scoring of GO terms

a Bar plots of GO term scores for 4 age groups related to immune cell specific activation. **b** Scores of T cell specific GO terms. **c** Scores of B cell specific GO terms. **d** Scores of GO terms of V(D)J recombinations.

Supplementary Fig. 7

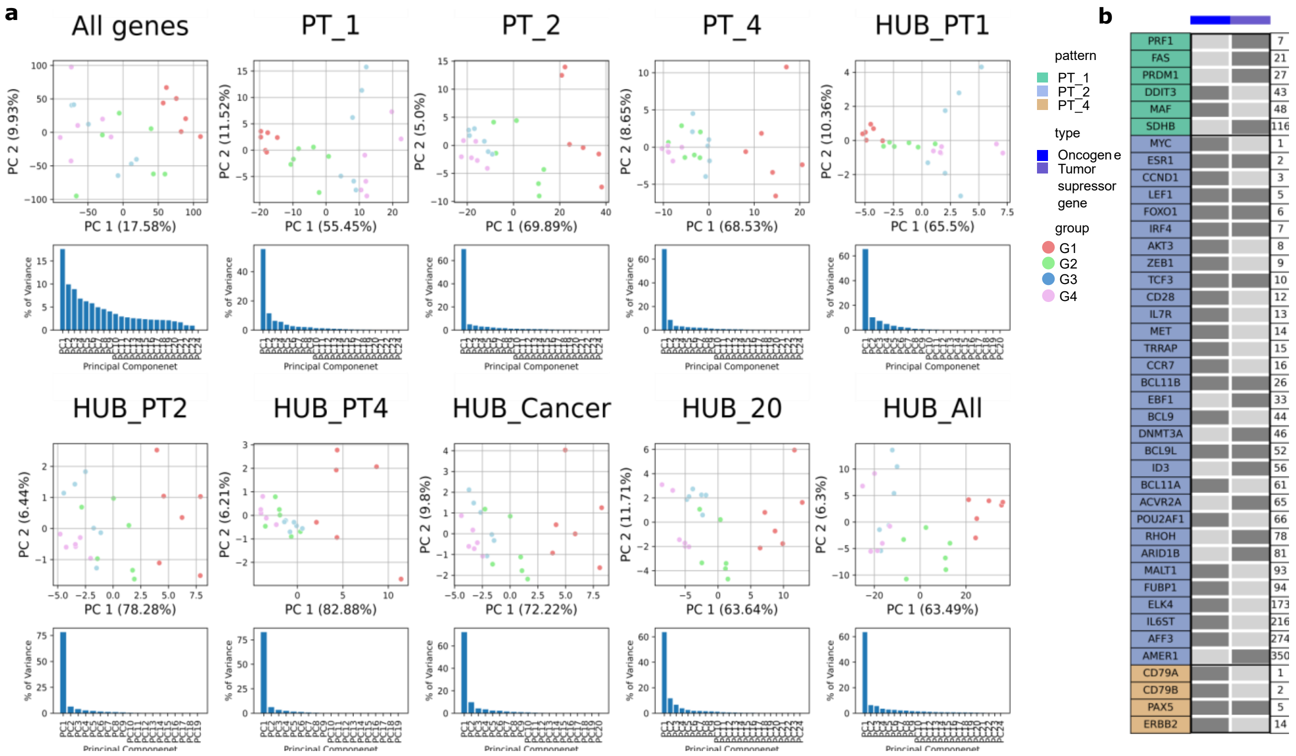

Supplementary Figure 7 : Principal component analysis (PCA) of analyzed group of genes.

a Principal components were analyzed with the group of genes All genes (n=27365), PT_1 (n=314), PT_2 (n=543), PT_4 (n=119), HUB_PT1 (n=20), HUB_PT2 (n=20), HUB_PT4 (n=20), HUB_19 (n=19), HUB_60 (n=60) and HUB_533 (n=533). Dot plot showing how the samples of each age group (G1:red, G2:green, G3:skyblue and G4:pink) are clustered well depending on the PC1 and PC2 of each groups. **b** 41 hub genes which have tumor regulation features among analyzed 533 hub genes of 3 patterns PT_1, PT_2 and PT_4. Blue and purple represent oncogene and tumor suppressor gene, and Black and grey color indicate Yes or No respectively.

Supplementary Fig. 8

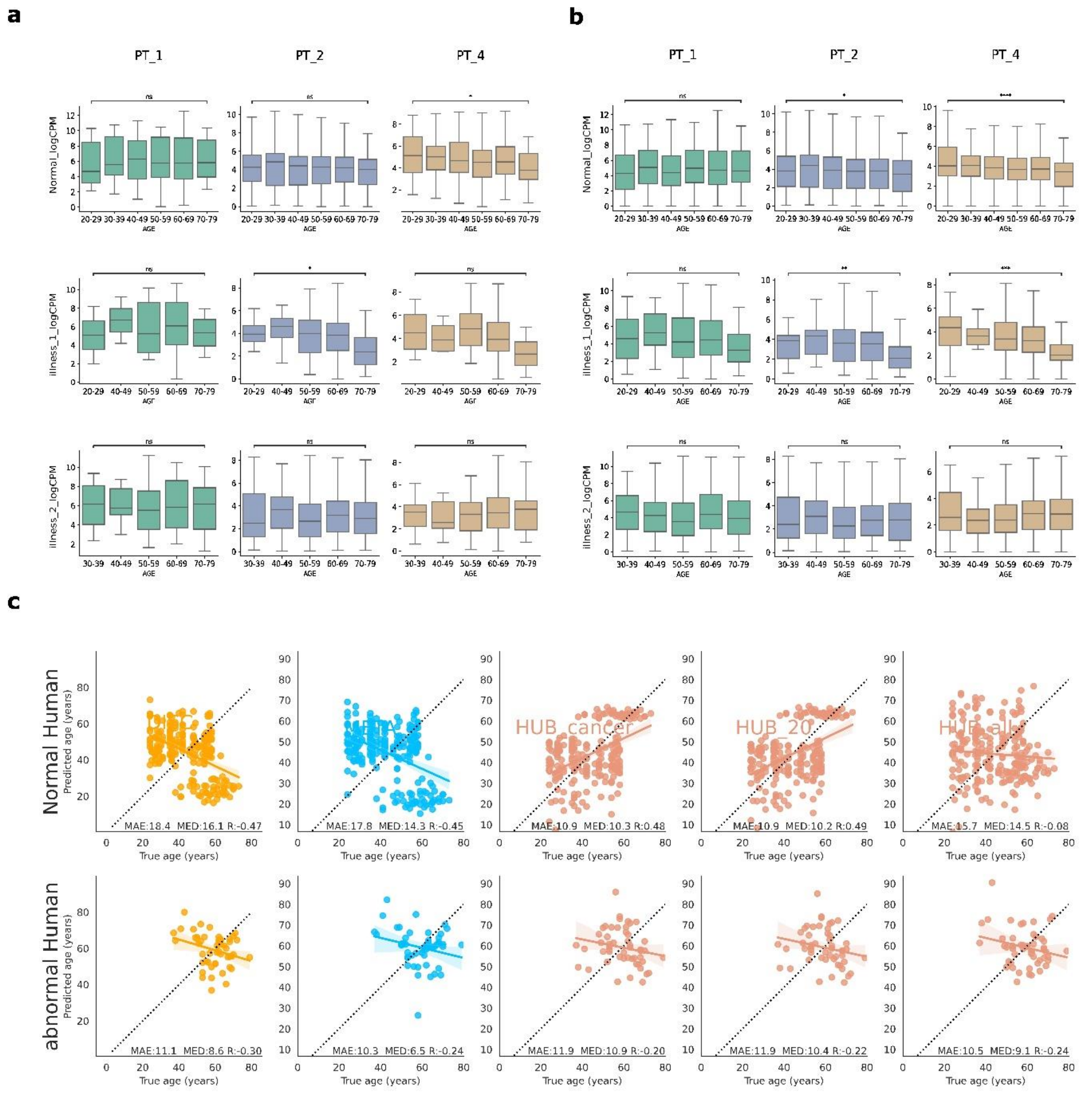

Supplementary Figure 8 : Aging-associated 3 patterns on human transcriptome data.

a GTEx expression patterns of 20 hub genes of selected patterns (PT_1, PT_2 and PT_4) that have tumor regulation features. Box plots were generated by logCPM value of the genes. Significant difference between the age group by two-tailed Mann-Whitney test with bonferroni correction were PT_4 of Normal (p-value = 1.292e-02) and PT_2 of illness_1 (p-value =

1.358e-02). **b** GTEx expression patterns of 59 hub genes (top 20 gene each) of the selected 3 patterns. Box plots were also generated by logCPM value. Significant difference by the Mann-Whitney test with bonferroni correction were PT_2 (p-value = 4.936e-02), PT_4 (p-value = 4.759e-05) of Normal, and PT_2 (p-value = 8.479e-03), PT_4 (p-value = 1.656e-04) of illness_1. **c** Scatter plot showing estimated age against true age on human transcriptomic data. logCPM values were used for the prediction. Transcriptomic age was estimated with the method introduced by previous studies the same as explained Fig.5b. The comparison was also evaluated with the metrics of mean absolute error (MAE), median absolute error (MED), Pearson's correlation (R).

Supplementary Fig. 9

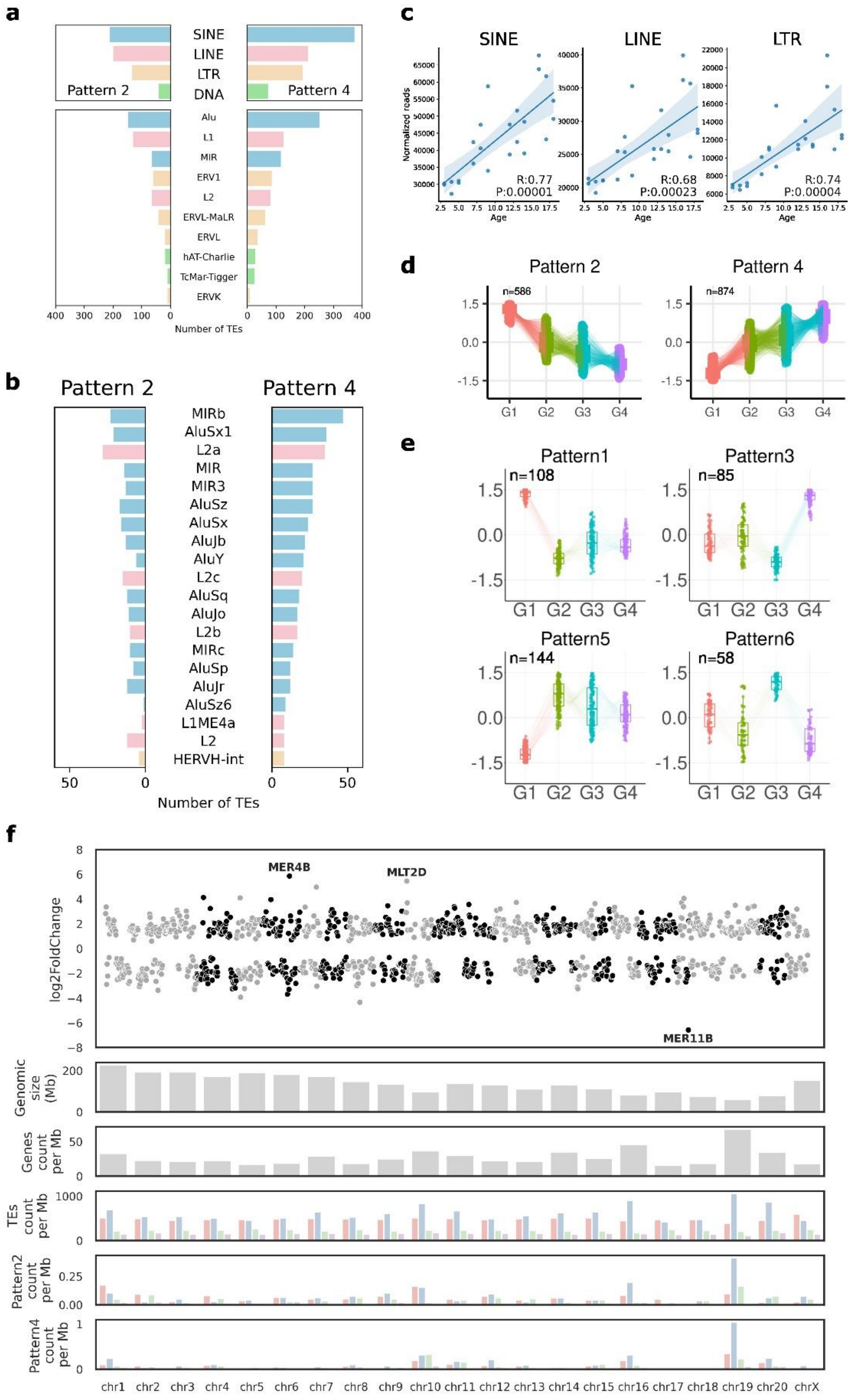

Supplementary Figure 9 : Transposable elements (TEs) expression

a Number of counts for superfamily and family of TEs. 2 bar plots left are for Pattern 2 and 2 bar plots right are for Pattern 4. Skyblue, pink, orange and green color indicate SINE, LINE, LTR, DNA respectively. **b** Number of counts for subfamily of TEs. Skyblue and pink indicate SINE, LINE respectively. **c** Line plot of normalized expression values of SINE, LINE and LTR family. Pearson's correlation analysis between these TEs and age was conducted. **d** Two significant DETEs patterns (Pattern2: n=586, Pattern4: n=874) analyzed by R package DEGreport with default setting but cutoff padj < 0.001. **e** The four rest DETEs patterns (Pattern1: n=118, Pattern3: n=89, Pattern5: n=146, Pattern6: n=63) identified by DEGreport with default setting but cutoff padj < 0.001. **f** Scatter plot depicting TE subfamily log_2 fold change (logFC) value on genome level. The subfamily names were indicated with |logFC| > 5. Bar plots showing genomic size, gene count, TE count, expressed TE count from Pattern 2 and Pattern with chromosome order.

Supplementary Table. 1

Sample	Sex	1st year Sampling		2nd Year sampling		3rd Year sampling	
		age	body weight (kg)	age	body weight (kg)	age	body weight (kg)
S1	male	3	2.02	4	2.5	5	2.89
S2	male	3	1.86	4	2.4	5	2.59
S3	male	7	4.14	8	4.42	9	4.59
S4	male	7	5.26	8	5.2	9	5.4
S5	male	12	4.18	13	4.32	14	4.46
S6	male	12	4.86	13	5	14	4.94
S7	male	16	4.66	17	5.1	18	4.88
S8	male	16	4.83	17	5.44	18	5.82

Supplementary Table 1 : Sample information.

All samples are from *Macaca fascicularis* called cynomolgus macaque or crab-eating monkey. Eight male individuals with two in one group were annually sampled. Body weight was also measured.
